# Can we use the internet to study speech production? Yes we can! Evidence contrasting online versus laboratory naming latencies and errors

**DOI:** 10.1371/journal.pone.0258908

**Published:** 2021-10-22

**Authors:** Amie Fairs, Kristof Strijkers

**Affiliations:** Laboratoire Parole et Langage, Aix-Marseille Université & CNRS, Aix-en-Provence, France; Universita degli Studi di Milano-Bicocca, ITALY

## Abstract

The closure of cognitive psychology labs around the world due to the COVID-19 pandemic has prevented in-person testing. This has caused a particular challenge for speech production researchers, as before the pandemic there were no studies demonstrating that reliable overt speech production data could be collected via the internet. Here, we present evidence that both accurate and reliable overt articulation data can be collected from internet-based speech production experiments. We tested 100 participants in a picture naming paradigm, where we manipulated the word and phonotactic frequency of the picture names. We compared our results to a lab-based study conducted on different participants which used the same materials and design. We found a significant word frequency effect but no phonotactic frequency effect, fully replicating the lab-based results. Effect sizes were similar between experiments, but with significantly longer latencies in the internet-collected data. We found no evidence that internet upload or download speed affected either naming latencies or errors. In addition, we carried out a permutation-style analysis which recommends a minimum sample size of 40 participants for online production paradigms. In sum, our study demonstrates that internet-based testing of speech production is a feasible and promising endeavour, with less challenges than many researchers (anecdotally) assumed.

## Introduction

One of the most common experimental paradigms to study speech production is picture naming, where participants come into the laboratory and name images presented on screen. These images are typically manipulated according to certain variables of interest, such as word frequency. This method of testing speech production has been fruitful for decades in uncovering aspects of word and sentence planning [1–5], and data from picture naming experiments has been vital for speech production theories [6–8]. However, with the current COVID-19 pandemic forcing labs to close, and social distancing and sanitary rules requiring extra measures even if participants are willing to come into a laboratory for in-person testing, researchers are considering internet-based studies of language production in order to collect data. While the online data collection for language perception is already well established [9–11], whether reliable and accurate speech production latencies can be collected online remains unclear, and this uncertainty makes speech production researchers hesitant to bring their paradigms out of the lab and onto the internet. For this reason, we carried out a speech production study, picture naming, deployed over the internet, to determine whether we could replicate the word frequency and phonotactic frequency effects commonly found in speech production [12, 13]. We had three additional goals above testing whether the frequency effect could be found: 1) whether the frequency effect and production latencies were similar to those of an in-person lab-based experiment, 2) whether participants’ internet speed affected the reaction times or error rates recorded, and 3) how many participants were necessary to test to establish the word frequency effect.

Outside of language production, some internet-based language comprehension and language learning studies have established that internet-based experiments can give reliable and robust data. These ‘mega studies’ of language processing have either recruited tens of thousands of participants [14, 15] or tested tens of thousands of linguistic items [10, 16, 17], and have demonstrated that it is possible to reliably collect a huge amount of data for psychological studies via the internet, which is almost impossible in a lab. However, it is not necessary to collect data from thousands of participants to obtain a reliable effect in online studies. Crump and colleagues [18] tested 10 different psychological paradigms both online (via Amazon’s Mechanical Turk) and in the lab. The paradigms tested included reaction time tasks, rapid stimulus presentation tasks, and learning tasks. For the reaction time and rapid presentation tasks, comparable performance was achieved between the online and lab samples of participants. However, for the learning tasks, the internet-based reaction times did not display the same pattern as the well-established lab-based studies. This implies that for some paradigms, potentially those which are more cognitively complex, it may not be possible to collect reliable data via the internet. Note also that audio recordings were not collected in any paradigms tested, hence the precision of audio recordings is unknown in these tasks.

One reason for why some paradigms may not be favourable for collecting reliable data via the internet is because of the inherent instability and uncertainty of a variety of technical factors, including the experimental program/programming language used, the operating system, the internet browser, the internet speed, and interactions between each of these factors. Recent studies [19, 20] have shown that there is inherent variability in all of these factors, which can affect the precision of the reaction time measured. For example, Anwyl-Irvine and colleagues (2020) show that both accuracy and variability in visual display and reaction times can vary, up to hundreds of milliseconds in some cases, according to different combinations of program, browser and operating system. They state “all platforms are reasonably accurate and reliable for studies not needing <100ms Reaction Time accuracy” (p. 19). However, in speech production experiments, we typically do need RT accuracy of less than 100ms, as effect sizes between experimental conditions can be much smaller than this. Bridges et al. (2020) found similar sized accuracy and variability in responses, and additionally investigated audiovisual synchrony when displaying both a visual and auditory stimulus concurrently. They found that while lab-based experimental software, such as EPrime, has submillisecond accuracy regardless of the operating system used, online software could have lags between the displays of the audio and visual stimuli of up to 100ms. These studies suggest that with a speech production paradigm, where visual information is displayed and an audio response recorded, reaction times collected could be very unreliable, with no way of concretely knowing if there are inherent precision issues in the quality of the recordings.

Aside from these issues, there is also a concern about the quality of the audio recordings and whether the microphones used at home by participants are good enough to collect data. All issues raised above may also interact with internet speed, such that there are differences in precision between participants based on their internet speed, or even within participants based on how taxed the upload and download components of their internet are. However, in a review of online studies, Hartshorne and colleagues [21] argue that effects which are greater than 30ms should be detectable in an internet-based experiment, as this effect size is large enough to deal with the technical variability possible in such experiments. Thus, if the size of the effect in the lab is greater than 30ms, as is the case with word frequency, it may be ‘immune’ to these technical issues.

We set out to test whether speech production data could reliably be collected via the internet. To our knowledge, only one other study has tested whether a speech production paradigm could be successfully carried out online, and this study was carried out at the same time as the experiment we report. Vogt et al. [22] tested whether the semantic interference effect could be accurately measured in a speech production experiment. In their study, the authors presented participants with an image with a distractor word written across the image. The distractor word could be semantically related or unrelated to the image, and the semantic interference effect refers to the fact that naming latencies are slower when naming a picture with a related distractor word printed across it compared to an unrelated distractor. They ran their experiments using the online experiment building language jsPsych [23] and preregistered their experiments with a sample size of 48 participants per experiment. The authors were able to detect the semantic interference effect when carrying out this experiment online, with an effect size of around 20ms, which is comparable to lab-based experiments [24]. This suggests that speech production experiments can be carried out reliably online, with similar effect sizes as in lab-based studies. Importantly, this study shows that effect sizes smaller than 30ms can be detected. Nevertheless, more internet-based speech production experiments are required to assess the robustness and reliability of overt articulation collected via the internet, to convince speech production researchers to take advantage of the many benefits that online testing can offer.

Though both studies were carried out in parallel independently from one another, luckily enough in the current study we tested a different speech production paradigm–picture naming–testing two different linguistic effects–word and phonotactic frequency. Word frequency is one of the most important psycholinguistic phenomena and relates to the frequency of occurrence of a word in a language, where people produce more frequent words faster than less frequent words [13]. While word frequency is a commonly manipulated variable, it shares some variation with another important psycholinguistic variable, age of acquisition, where people produce earlier acquired words faster than later acquired words, and there is a strong relationship between the two variables [25]. In studies where both word frequency and age of acquisition are controlled, evidence for both phenomena can be found [26], but sometimes only age of acquisition effects are significant [27]. Phonotactic frequency relates to how frequently two different syllables occur together. Similarly to word frequency, highly frequent syllable pairs are produced faster than less frequent syllable pairs [28, 29]. We aimed to directly replicate a lab-based experiment [30], by using the same stimuli and procedure in the internet study as in the lab study. Our main aim was to determine if we could detect a word frequency effect. The word frequency effect is strongly robust in psycholinguistic studies, and if this could not be detected online there would be serious concerns over the reliability of internet-based production data. In addition, we explored whether a phonotactic frequency effect would be detected. In Fairs et al., a significant word frequency effect was found, but the phonotactic frequency effect was not significant. While the word frequency effect is strongly robust in the literature, the phonotactic frequency effect is much more subtle and unstable, with studies finding the effect in one experiment but not in another [31, 32]. This offers an interesting contrast for the current study where we test both the robust variable of word frequency, and the less stable (and sometimes elusive) variable of phonotactic frequency. In addition, we a) sought to directly compare the results of Fairs et al. with the results obtained via the internet study to characterise the pattern of speech production latencies when collected in an internet study, especially if different to those from the lab; b) determine whether the internet upload and download speed of participants had an effect on naming latencies or errors; and c) calculate the sample size of participants necessary to establish a significant effect, calculated by randomly sampling different numbers of participants from our data, running our analyses multiple times, and calculating how often the word and phonotactic frequency effects were significant. We describe the study and later discuss practical recommendations and hurdles that challenged us along the way.

## Methods

All materials, data (except the raw audio recordings due to privacy concerns), and analyses are available on the OSF at https://osf.io/hncf4.

### Participants

100 native French-speaking participants took part in the study (of the 79 participants who provided demographic data: 63 female, 15 male, 1 declined to answer; age = 25.7 years, SD = 3.8 years), recruited through the participant database at the Laboratoire Parole et Langage, Aix-Marseille Université. Participants self-reported as right-handed, with no language or neurological disorders, and normal or corrected-to-normal vision. Participants were paid €10 for participation and the study was carried out in line with ethical considerations of the Declaration of Helsinki. We a-priori planned to test 100 participants, which is a larger sample size than many speech production experiments (where the typical range is between 20 and 40 participants), to enable us to determine a sample size of participants to produce a significant effect. Data from 29 of the 100 participants were not analysed due to no audio data being recorded (1 participant), microphone quality being too bad to determine the words spoken (1 participant), participants using an article before the picture name (2 participants), participants not fulfilling recruitment criteria (2 participants, both left-handed), and ‘bad-faith’ participants (22 participants). We discuss participant uptake and rejection in greater detail in the General Discussion.

### Design & materials

The design and materials were very similar to the production experiment reported in Fairs, Michelas, Dufour & Strijkers (2021), except the current study was run online.

Participants took part in a picture naming experiment, where coloured line-drawn images from the MultiPic database [33] were presented on screen. All images were sized 300 by 300 pixels and presented on a white background. Target stimuli varied along two within-participant experimental variables: word frequency and phonotactic frequency. Values for these manipulations were retrieved from the Lexique database [34]. Log word frequency values ranged from 0 to 2.88, with a mean low log frequency of 0.5 and a mean high log frequency of 1.54. Phonotactic frequency (the summed syllable frequency of the target words) values ranged from 87 to 14089, with a mean low phonotactic frequency of 2052.5 and a mean high phonotactic frequency of 6836.56. These variables were orthogonally manipulated, with 55 items per cell, leading to 220 target items overall. Target stimuli were additionally controlled for their h-index value (the number of alternative names given to the image presented for that target), visual complexity, bigram frequency, number of phonological neighbours, phoneme length, and phoneme frequency (all comparisons are reported in the OSF repository), but items were not controlled for age of acquisition.

In addition to the target stimuli, 25 filler stimuli were presented. These were all food items, and these trials were not analysed. Filler trials were included to keep the design as similar as possible to Fairs et al., where these same stimuli were used in both a production and perception experiment (in the perception experiment, participants pressed a button when food items (the filler trials) were presented). In total, 245 images were shown to participants to name. An additional 6 images were used for practice trials.

The 245 trials were split into 5 blocks which were balanced overall by high vs low word and phonotactic frequency. Per participant, trials within each block were randomised and the order of the blocks was randomised.

### Procedure

The experiment was run online using the experimental platform FindingFive [35], which allows experiments to be deployed via the internet from FindingFive’s server. Participants ran the experiment using a web browser. Before taking part in the experiment, participants created a FindingFive account (and a PayPal account, if needed, to receive payment after the experiment was completed). Participants began the experiment by reading through the consent form and general experiment instructions. Participants were then explicitly asked to type that they had read the consent form and would take part in the study. Demographic variables for participants were collected, such as their date of birth, gender, handedness, if they suffered from any language disorders, native language, and any other languages they spoke (and their level of proficiency). While there were study-specific recruitment criteria (e.g., participants should be native French speakers, between the ages of 18 and 40, right-handed, with normal or corrected-to-normal vision, and with no language or neurological disorders), this could not be verified before beginning the experiment in the same way as can be done in person in the lab. Therefore, we collected this demographic data as a check to then remove participants’ data from analysis if participants did not fulfil the study criteria and took part anyway. For two participants this was the case (both reported being left-handed).

We also collected technical information from the participants: the browser the experiment was running on, the upload and download speed of the participant’s internet (participants visited the site www.speedtest.net and reported their upload and download speeds), the internet ‘type’ (e.g., fiberoptic, cable), the make and model of their laptop/computer, and whether participants were using external microphones/speakers. These variables were collected in case we discovered technical problems in the recording. Participants’ download and upload speeds were added as variables into the analysis.

After participants provided this information, they began the experiment with 6 practice trials before moving on to the experiment proper. One each trial, a fixation cross (500ms) was displayed, followed by a blank screen (500ms), a picture (630ms), and a final blank screen (of roughly 1420ms). This final blank screen varied in duration as it was composed of two ‘different’ blank screens (the screen continually stayed blank from the perspective of the participants). The duration of this screen was automatically recorded by FindingFive. The first blank screen timed out after roughly 2000ms (mean = 2007.9s, SD = 127ms, range = 21ms to 20707ms) after picture display onset, and during this time (picture presentation + blank screen up to 2000ms) the picture name audio responses were recorded. All audio responses lasted 1998ms. Following this, a second blank screen was presented, which was designated as the time the trial data should be sent back to the FindingFive server for recording during the experiment. In general, this took around 57.7ms (SD = 37ms, range = 7ms to 910ms). The experiment was designed in this way such that the fixation cross and picture presentation would always be displayed for a fixed amount of time, and we assumed that the only internet-signal-related variation in timing would be present after the response was recorded (i.e., in the second blank screen).

Trials were presented in blocks of 49 trials, with a break of 10 seconds between blocks. At the end of the experiment participants were asked to comment on anything strange during the experiment, asked to give their email addresses if they would want to be contacted with the results of the study, and fully debriefed. In total, including creating PayPal and FindingFive accounts, the experiment lasted approximately 40 minutes.

### Data analysis

Speech responses were recorded in individual files and saved as.ogg files by the experimental software. These files were converted to.wav files using Human Media Audio Converter (version 1.9.7) and annotated semi-automatically using a custom-made script in Praat [36] to calculate speech latencies, and check for errors.

Errors were defined as any trial where participants did not respond, produced a name different to the target name, hesitated, coughed or otherwise produced a disfluency before naming the target, or where there was noise in the recording such that the onset of speech could not be accurately determined. These trials were removed from the main analysis of speech latencies. We did not set a minimum number of trials necessary to retain participants for analysis. Across all participants, 68% of data remained for the main latency analysis (ranging from 11% to 81% by participants), with 32% included in the error analysis.

Latencies were analysed using linear mixed effects models and errors were analysed with generalised linear mixed effects models, both from the lme4 package [37] in R [38]. All continuous variables were centred and scaled. All categorical variables used sum-to-zero contrast coding. Production latencies were log-transformed for analyses to reduce skew. Errors were modelled as binomial with a logit link function. All models were fit with the maximal random structure which would converge and did not result in a singular fit, and each model is reported below. T values from the models are reported, where we take t values greater than |2| to be significant. For the error analyses, p values are also reported as they are provided by the glmer output. Confidence intervals and tables were generated from sjPlot [39], where the tables were further edited, and data are displayed using plotting functions from ggplot [40].

## Results

### Production latency analyses

**Fig 1:**
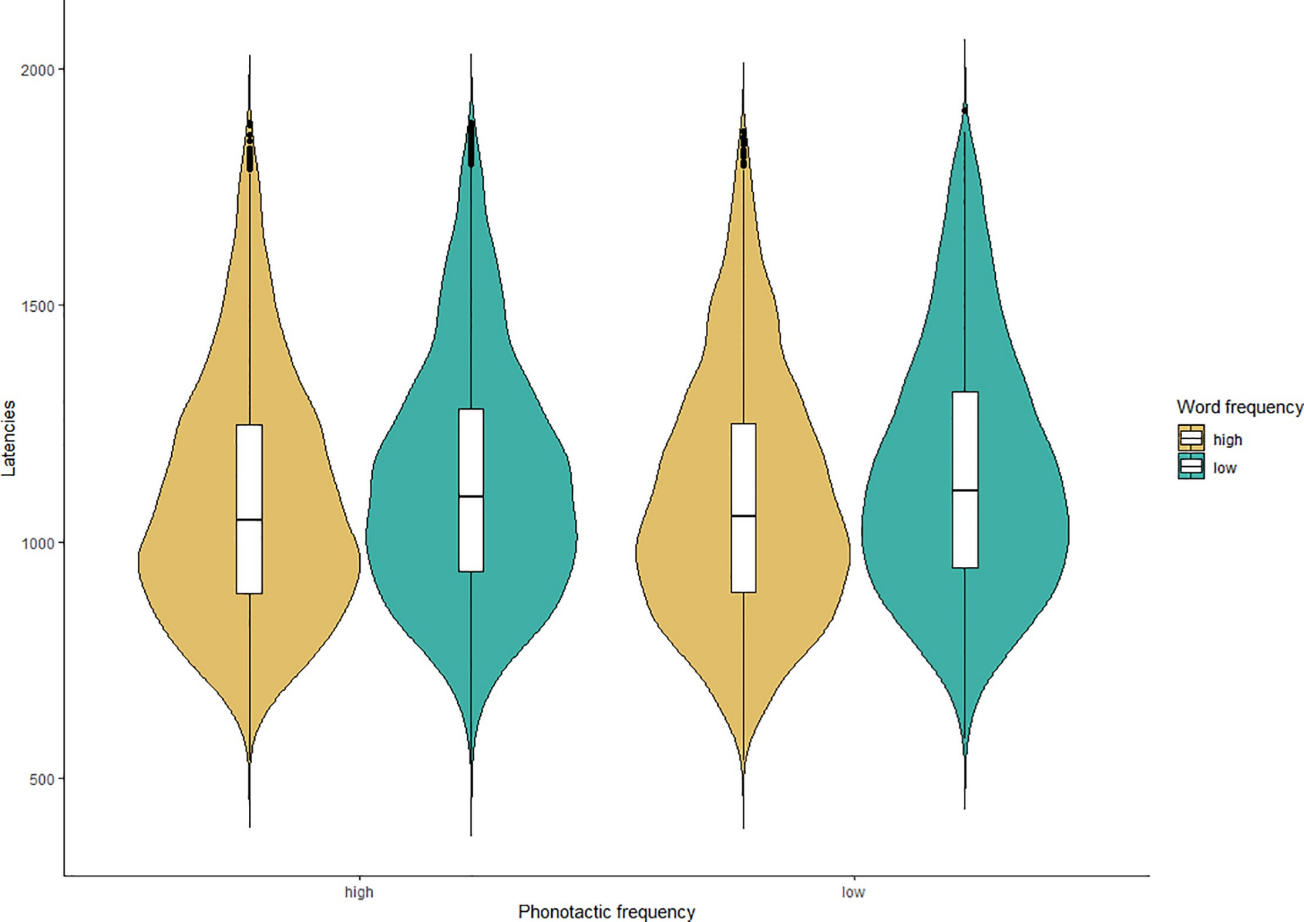
Naming latency distributions. Latencies by word frequency (high: yellow, low: green) and phonotactic frequency (high: left, low: right).

For items with high word frequency, mean production latencies were 1085.4ms (SD = 300ms), and for low frequency items RTs were 1136.8ms (SD = 312ms), with a difference of 51ms. For items with high phonotactic frequency, mean production latencies were 1105ms (SD = 306ms) and for items with low phonotactic frequency latencies were 1114.1ms (SD = 309ms), with a difference of 9ms (see Fig 1).

Our first question was whether we would find significant effects of word and phonotactic frequency. To answer this, latencies were analysed with linear mixed effects models with the fixed effects of word frequency, phonotactic frequency (both categorical variables), and their interaction, the covariate predictor trial (the trial number), and random structure containing random intercepts by participants and items, and a random slope of word frequency by participant. We found a significant main effect of word frequency, but no significant effect of phonotactic frequency, nor their interaction (see Table 1). We ran the same model structure but with continuous predictors of word and phonotactic frequency, and found the same pattern of results (see Table 1). We additionally ran both models on raw latencies, rather than log-transformed, to ensure the log-transformation did not skew the results. We found the same pattern of results in both models on raw latencies, though the random slope in the categorical model on raw latencies was removed due to non-convergence. These results suggest that in an online study with these materials we can replicate the word frequency effect, but we do not have evidence for the phonotactic frequency effect.

**Table 1:** Linear mixed model outputs of latency analyses.

	Categorical frequency predictors			Continuous frequency predictors		
*Predictors*	*Estimates*	*CI*	*Statistic*	*Estimates*	*CI*	*Statistic*
(Intercept)	7.0255	6.9908 – 7.0603	396.1627	7.0234	6.9886 – 7.0583	395.1225
Trial	0.0048	0.0017 – 0.0078	**3.0742**	0.0048	0.0018 – 0.0078	**3.0994**
Word freq	-0.0264	-0.0426 – -0.0102	**-3.1981**			
Phonotactic freq	-0.0049	-0.0209 – 0.0111	-0.6038			
Word*phonotactic freq	-0.0000	-0.0160 – 0.0160	-0.0051			
Log word freq				-0.0285	-0.0444 – -0.0125	**-3.5011**
Log phonotactic freq				0.0053	-0.0105 – 0.0211	0.6539
Log word*phonotactic freq				-0.0015	-0.0168 – 0.0139	-0.1859
**Random Effects**						
Residual variance	0.0246			0.0245		
Item intercept	0.0139			0.0138		
Participant intercept	0.0176			0.0177		
Word freq by participant	0.0001			0.0001		
Correlation between participant intercept and word freq	0.6010			0.5492		
N	71 _participant_			71 _participant_		
	219 _item_			219 _item_		
Observations	10542			10542		

Statistics are t values, and those in bold are above the significance threshold of |2|. Freq: frequency.

Our items were not balanced by the sonority of their onset phoneme, which can have an effect on naming latencies (thank you to an anonymous reviewer for bringing this to our attention; [41, 42]). We thus coded all items by the sonority of their onset phoneme (following [43]) and added sonority to both the continuous and categorical frequency models. We found no effects of sonority as either a main effect or in interaction with the frequency variables in either model (all t’s < 2; see the OSF repository for the model outputs), but a main effect of word frequency was still found. This suggests that even though the sonority of the onset phoneme was not balanced across conditions in this study, sonority did not affect naming latencies.

Our second question was whether these results were significantly different from the lab-based experiment reported in Fairs et al., which this internet study was modelled on, which contained a word frequency effect of 68ms and phonotactic frequency effect of 1ms. To test this, we combined the latencies from this internet study and the lab study. Our model contained fixed effects of word frequency, phonotactic frequency, experiment (internet or lab), and their interactions, the covariate predictor trial number, and random intercepts by participant and by item. We found significant effects of word frequency and experiment, and a significant interaction between word frequency and experiment (see Table 2).

**Table 2:** Linear mixed model outputs of latency analyses across the internet and lab experiments.

	All data			Lab data trimmed to max 2000ms		
*Predictors*	*Estimates*	*CI*	*Statistic*	*Estimates*	*CI*	*Statistic*
(Intercept)	6.9427	6.9051 – 6.9803	361.7221	6.9348	6.8977 – 6.9720	365.8908
Trial	0.0107	0.0075 – 0.0139	**6.5450**	0.0100	0.0069 – 0.0131	**6.2966**
Word freq	-0.0293	-0.0457 – -0.0129	**-3.4999**	-0.0264	-0.0423 – -0.0104	**-3.2380**
Phonotactic freq	-0.0018	-0.0182 – 0.0146	-0.2171	-0.0026	-0.0186 – 0.0133	-0.3223
Experiment	-0.0897	-0.1238 – -0.0555	**-5.1502**	-0.0959	-0.1297 – -0.0621	**-5.5587**
Word*phonotactic freq	0.0046	-0.0118 – 0.0210	0.5532	0.0047	-0.0112 – 0.0207	0.5814
Word freq*experiment	-0.0047	-0.0089 – -0.0005	**-2.1717**	-0.0013	-0.0054 – 0.0028	-0.6111
Phonotactic freq*experiment	0.0016	-0.0026 – 0.0058	0.7471	-0.0001	-0.0042 – 0.0040	-0.0403
Word freq*phonotactic freq*experiment	0.0019	-0.0023 – 0.0061	0.8894	0.0029	-0.0012 – 0.0071	1.4096
**Random Effects**						
Residual variance	0.0346			0.0325		
Item intercept	0.0164			0.0155		
Participant intercept	0.0169			0.0166		
N	89 _participant_			89 _participant_		
	252 _item_			252 _item_		
Observations	13327			13273		

Statistics are t values, and those in bold are above the significance threshold of |2|. Freq: frequency.

**Fig 2:**
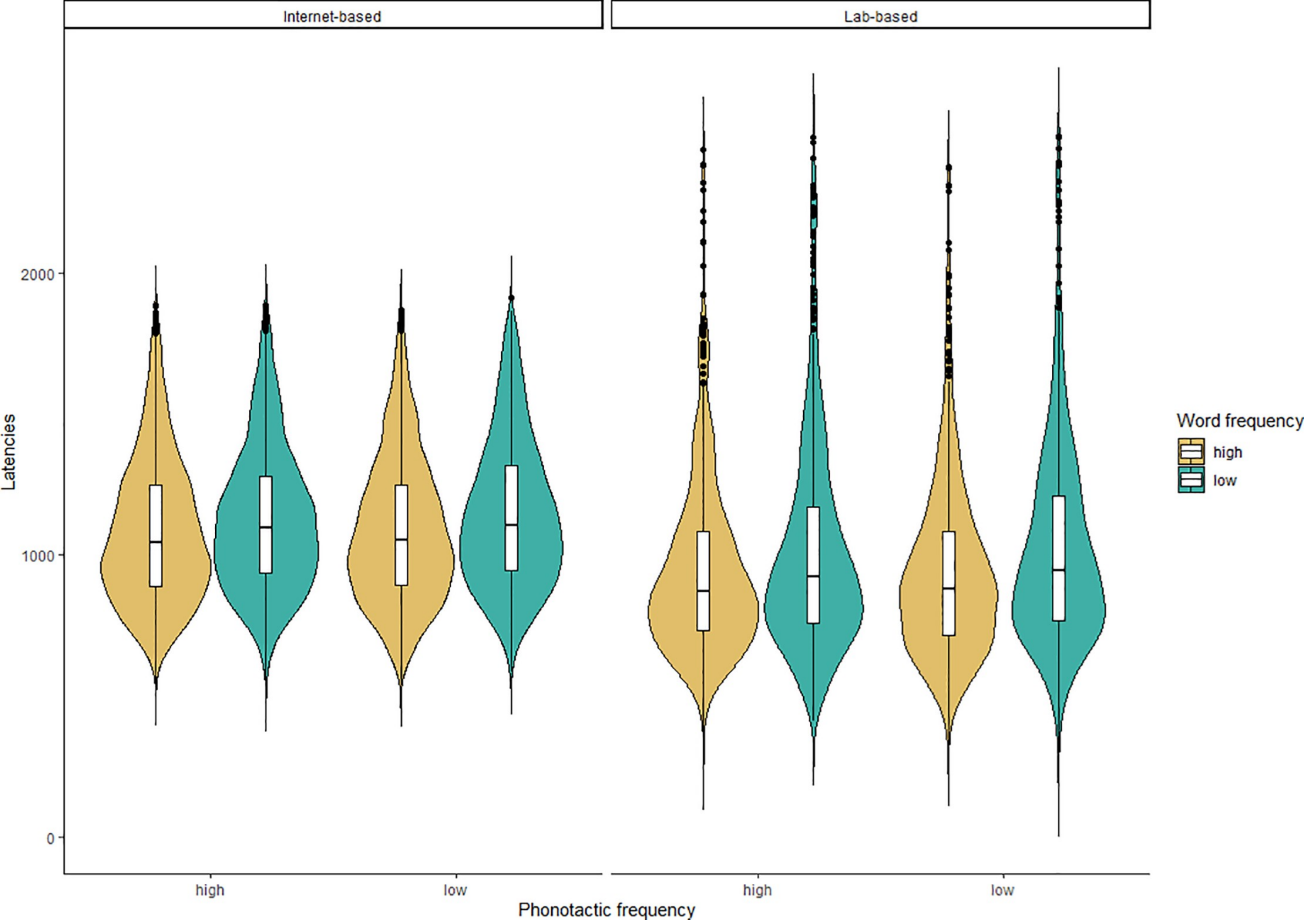
Naming latency distributions across experiments. Latencies for the internet-based experiment (left panel) and the lab-based experiment (right panel), by word frequency (high: yellow, low: green) and phonotactic frequency (within each panel, high: left, low: right).

However, a closer inspection of the data revealed that there were slightly different ‘cut off’ values for the end of a trial in both experiments. As can be seen in Fig 2, in the internet experiment latencies cannot exceed 2000ms because the audio recordings only lasted 1998ms after the onset of the image. If participants spoke after this time, i.e., more than 2000ms from the onset of the image, it would not be recorded. For the lab-based study, while the trials were the same length it was possible for the voicekey to trigger later in some instances, where some latencies were above 2000ms. To make a fairer comparison between the internet and lab study, we thus removed latencies in the lab-based experiment which were above 2000ms and re-ran the model with the same structure. These results indicated a significant effect of word frequency and a significant effect of experiment, but no significant interaction. This is in line with the average word frequency effect size of 56ms in the lab data when latencies larger than 2000ms are excluded. Again, when running these models on raw latencies, the same pattern of results was found. These results suggest that the frequency effect is of comparable magnitude when collected via the internet versus when collected in the lab. However, the latencies are significant slower when collected via the internet. We discuss possible reasons for this in the General Discussion.

Our third question was whether internet speed affected latencies. Providing internet speed was optional for participants and 39 participants provided this information. Mean download speed was 145mb (SD = 244.7, range: 0.61mb to 1000mb). Mean upload speed was 85mb (SD = 126.6, range: 0mb to 415mb). To test our question, our model contained fixed effects of word frequency, phonotactic frequency, internet upload speed, internet download speed, and interactions between upload speed, word frequency and phonotactic frequency, and between download speed, word frequency and phonotactic frequency. The covariate predictor trial was also entered into the model. The random structure contained random intercepts by participant and item, and a random slope of word frequency by participants. The only significant effect in the model was word frequency; upload and download speed had no significant effects and did not significantly interact with the frequency variables (Table 3). We found the same pattern of results when running the same model structure with continuous predictors of word and phonotactic frequency (see Table 4). When running the same model structures on raw latencies, we again found the same pattern of results as with log-transformed latencies. Thus, in these models, we do not have any evidence that internet upload or download speed affected production latencies, nor interacted with our frequency variables.

**Table 3:** Linear mixed model outputs of internet speed affecting latency, with categorical frequency predictors.

	Categorical frequency predictors		
*Predictors*	*Estimates*	*CI*	*Statistic*
(Intercept)	7.0018	6.9536 – 7.0499	284.9024
Trial	-0.0017	-0.0058 – 0.0024	-0.8083
Upload speed	-0.0217	-0.0826 – 0.0392	-0.6980
Word freq	-0.0271	-0.0439 – -0.0103	**-3.1613**
Phonotactic freq	-0.0053	-0.0219 – 0.0112	-0.6342
Download speed	-0.0380	-0.1073 – 0.0313	-1.0740
Upload speed*word freq	-0.0011	-0.0079 – 0.0058	-0.3062
Upload speed*phonotactic freq	0.0016	-0.0039 – 0.0070	0.5637
Word*phonotactic freq	0.0014	-0.0151 – 0.0179	0.1672
Word freq*download speed	-0.0035	-0.0111 – 0.0042	-0.8945
Phonotactic freq*download speed	-0.0022	-0.0083 – 0.0038	-0.7182
Upload speed*word freq*phonotactic freq	0.0032	-0.0022 – 0.0086	1.1569
Download speed*word freq*phonotactic freq	-0.0007	-0.0068 – 0.0054	-0.2261
**Random Effects**			
Residual variance	0.0254		
Item intercept	0.0143		
Participant intercept	0.0206		
Word freq by partciipant	0.0001		
Correlation between participant intercept and word freq	0.4062		
N _participant_	39		
N _item_	217		
Observations	6009		

Statistics are t values, and those in bold are above the significance threshold of |2|. Freq: frequency

**Table 4:** Linear mixed model outputs of internet speed affecting latency, with continuous frequency predictors.

	Continuous frequency predictors		
*Predictors*	*Estimates*	*CI*	*Statistic*
(Intercept)	6.9997	6.9515 – 7.0479	284.4748
Trial	-0.0016	-0.0058 – 0.0025	-0.7827
Download speed	-0.0383	-0.1077 – 0.0311	-1.0803
Log word freq	-0.0294	-0.0461 – -0.0127	**-3.4556**
Log phonotactic freq	0.0053	-0.0110 – 0.0216	0.6326
Upload speed	-0.0217	-0.0828 – 0.0393	-0.6977
Download speed*Log word freq	-0.0054	-0.0138 – 0.0030	-1.2647
Download speed*Phonotactic freq	-0.0012	-0.0073 – 0.0048	-0.3984
Log word freq*log phonotactic freq	-0.0011	-0.0171 – 0.0150	-0.1295
Log word freq*upload speed	-0.0012	-0.0086 – 0.0063	-0.3036
Log phonotactic freq*upload speed	0.0036	-0.0018 – 0.0091	1.3018
Download speed*log word freq*log phonotactic speed	-0.0017	-0.0080 – 0.0047	-0.5172
Upload speed*log word freq*log phonotactic freq	0.0029	-0.0027 – 0.0085	1.0083
**Random Effects**			
Residual variance	0.0253		
Item intercept	0.0141		
Participant intercept	0.0207		
Word freq by participant	0.0001		
Correlation between participant intercept and word freq	0.3907		
N _participant_	39		
N _item_	217		
Observations	6009		

Statistics are t values, and those in bold are above the significance threshold of |2|. Freq: frequency

As explained in the methods section, we had a specific blank screen on each trial where the trial-level data was sent to the FindingFive server. We correlated the average length of this blank screen per participant with the upload and download speed values given by participants to determine whether the length of the screen varied based on internet speed. Neither the upload speed by blank screen duration correlation (t(37) = 0.89, r = 0.14, p = 0.38) nor the download speed by blank screen duration correlation (t(48) = 1.28, r = 0.18, p = 0.2) was significant.

Our fourth question was how many participants were necessary to detect an effect. To answer this question, we chose a permutation-style approach. We randomly selected all data from a certain number of participants (e.g., 20 participants) and ran a model on this subset, which contained fixed effects of word and phonotactic frequency and their interaction, the covariate predictor trial, and a random structure including random intercepts by participant and item, and a random slope of word frequency by participant. We also calculated the mean frequency effect sizes and SDs in latencies for this smaller subset. We repeated this participant selection and modelling procedure 1000 times. We then inspected the model outputs and calculated how often, as a percentage of the 1000 runs, the t values for the word and phonotactic frequency effects were greater than |2|. We also averaged the effect sizes for word and phonotactic frequency across runs. The final column displays the raw number of singular fit warnings given by the models. A singular fit is not a problem statistically but can affect generalizability of any interpretations, because the model is ‘overfit’ [44, 45]. Table 5 displays all results.

**Table 5:** Permutation approach to determine a sample size of participants necessary to detect a frequency effect.

Number of participants	Sig. word freq (%)	Sig. phonotactic freq (%)	Mean word freq effect (ms)	SD word freq effect (ms)	Mean phonotactic freq effect (ms)	SD phonotactic freq effect (ms)	Number of singular fit warnings
5	63	3	51	65.6	10.2	70.1	616
10	91.3	0	51.2	42.6	9.6	49.4	379
15	98.5	0	51.7	34.4	9.8	42.7	285
20	99.6	0	51.7	28.6	9.7	38.1	190
25	100	0	51.3	24.2	9.9	34.8	130
30	100	0	51.7	21.1	10	32.9	91
35	100	0	51.5	18.6	9.9	31.3	54
40	100	0	51.4	16.4	9.9	29.9	33
45	100	0	51.4	14.5	9.7	29	15
50	100	0	51.5	12.5	9.9	28	3
55	100	0	51.5	11	9.8	27.4	3
60	100	0	51.4	9.2	9.9	26.7	0

The ‘Sig. word freq’ and ‘Sig phonotactic freq’ columns display the percentage of times the word or phonotactic frequency effect was significant in the 1000 runs. Freq: frequency.

We found that with just 10 participants the word frequency effect was significant more than 90% of the time, and increasing the number of participants to 25 resulted in the word frequency effect being significant 100% of the time, i.e., in all of the 1000 models run. The phonotactic frequency effect was almost never significant, and the standard deviations of mean phonotactic frequency effect were always large. If we were to make sample size claims based on the word frequency effect alone, it would suggest that 25 participants would suffice. However, while the mean word frequency effect size stays fairly stable throughout different numbers of participants, the standard deviation does not. With only 25 participants, the standard deviation is around half the size of the effect. In addition, of the 1000 models run containing data from 25 participants, 130 of these models resulted in a singular fit, meaning that 13% of the models were overfit and not generalizable to the wider population.

Based on these results, we suggest that a sample size of 40 participants would be optimal for an effect of 50ms. With 40 participants, we found the standard deviation to be low, and while subjective, a 3.3% chance of obtaining a singular fit is seemingly an acceptable threshold. Remember that we randomly selected participants from our pool of 71, and some of these participants had low data retention rates, meaning it is possible there was not enough data in some of these models to ‘prevent’ a singular fit. While this analysis demonstrates that more data is always better, we would argue that with 40 participants the effects are stable, and the cost and time taken to recruit and analyse a larger sample size of participants is not offset by a greater chance to detect a significant effect.

### Error analyses

**Fig 3:**
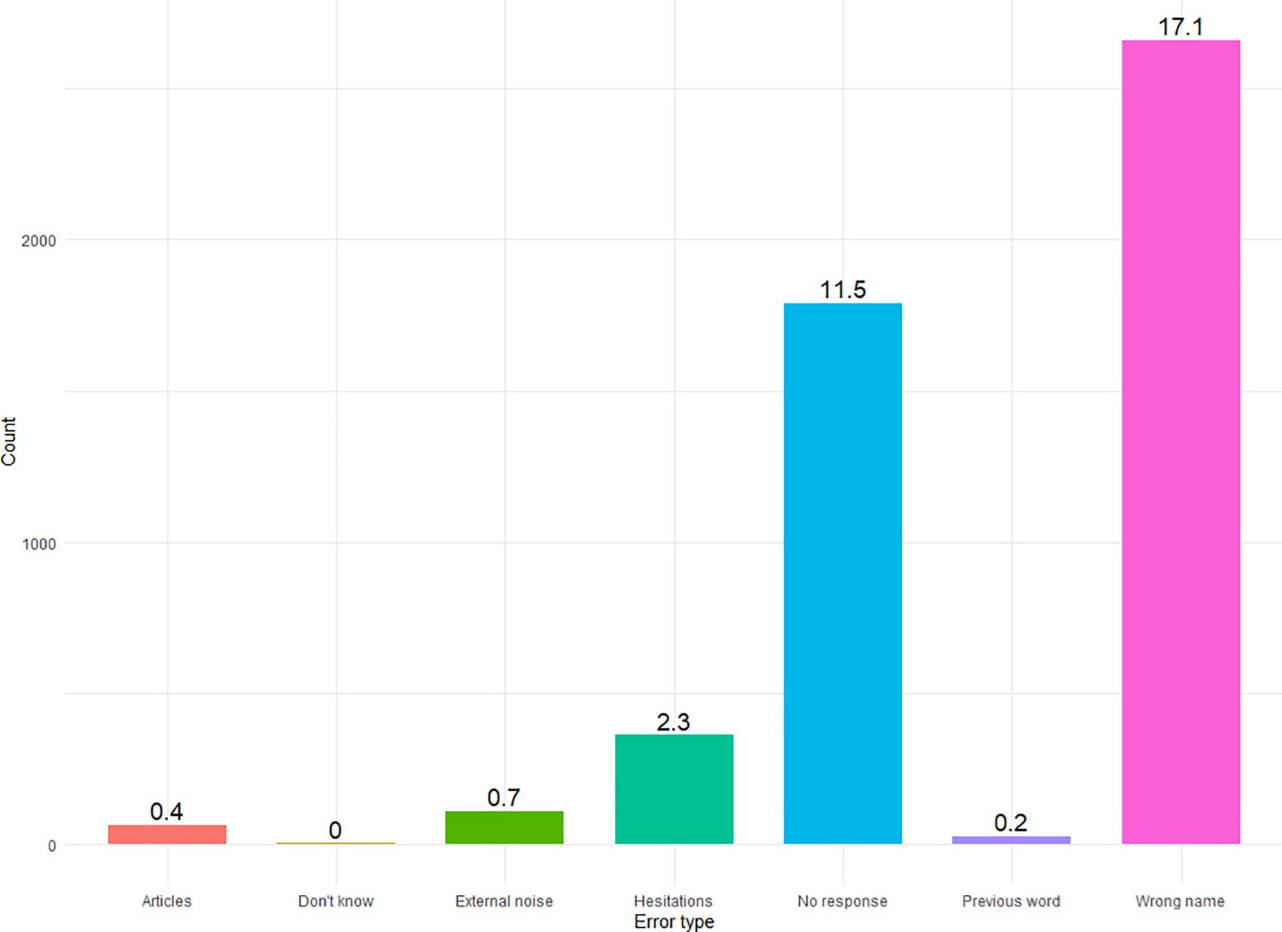
Errors. Raw counts and percentages (in text above the bars) of error types. ‘Articles’ = participants produced ‘a’ or ‘the’ before the noun; ‘Don’t know’ = participants said ‘I don’t know’; ‘External noise’ = external noise in the recording inhibited detection of speech onset; ‘Hesitations’ = participants hesitated before producing a word; ‘No response’ = no response was recorded; ‘Previous word’ = participants named the image on the previous trial; ‘Wrong name’ = participants provided the wrong name of the picture.

Overall, 32% of all analysable data were errors, displayed in Fig 3, where on the y axis we display raw counts of errors and the numbers above the bars are percentages of that error type from all data. Descriptively, 32% errors is quite high, and we discuss reasons for this in the General Discussion. We see that the highest number of errors with 17% were in the ‘wrong name’ category, where participants provided an acceptable but incorrect name for an image, such as ‘postier’ (*postman*, dialectal) for ‘facteur’ (*postman*), or ‘manchot’ (a type of *penguin*) for ‘pingouin’ (*penguin*). The next highest error type, with 11.5% of the data of this type, was ‘no response’, where silence was recorded on the trial.

Our last question was whether both our frequency variables and internet speed affected errors. To test this question, our model contained fixed effects of word frequency, phonotactic frequency, internet upload speed, internet download speed, and interactions between upload speed, word frequency and phonotactic frequency, and between download speed, word frequency and phonotactic frequency. The covariate predictor trial was also entered into the model, and the random structure contained random intercepts of participant and item. We found a significant effect of word frequency, such that there were more errors for low frequency items than high frequency items. There were no main effects of internet speed, and the only interaction which reached significance was upload speed interacting with phonotactic frequency. Upload speed had no effect for high phonotactic frequency items, but for low phonotactic frequency items the faster the upload speed the more errors there were (see Table 6). This is an unexpected and slightly bizarre finding, and we do not have an explanation for the implications of this effect. Because each participant provided their individual upload speed, hence upload speed is to some extent a proxy for participant number, it could be that for one participant there was a strong phonotactic frequency effect in the errors, which comes out in this interaction. Running the same model structure with continuous frequency values would not converge.

**Table 6:** Generalised linear mixed model output on error proportions.

	Errors			
*Predictors*	*Odds Ratios*	*CI*	*Statistic*	*p*
(Intercept)	3.44	2.56 – 4.62	8.21	**<0.001**
Trial	1.04	0.98 – 1.10	1.33	0.184
Upload speed	0.92	0.69 – 1.23	-0.53	0.593
Word freq	1.29	1.05 – 1.58	2.39	**0.017**
Phonotactic freq	1.01	0.82 – 1.24	0.11	0.915
Download speed	1.15	0.83 – 1.59	0.85	0.394
Upload speed*word freq	1.02	0.94 – 1.10	0.48	0.629
Upload speed*phonotactic freq	1.11	1.03 – 1.19	2.64	**0.008**
Word*phonotactic freq	0.98	0.80 – 1.21	-0.15	0.877
Download speed*word freq	0.99	0.91 – 1.08	-0.12	0.905
Download speed*phonotactic freq	0.96	0.89 – 1.05	-0.83	0.405
Upload speed*word freq*phonotactic freq	0.98	0.91 – 1.06	-0.40	0.690
Download speed*word freq*phonotactic freq	0.97	0.89 – 1.06	-0.59	0.553
**Random Effects**				
Residual variance	3.29			
Item intercept	2.23			
Participant intercept	0.44			
N _participant_	39			
N _item_	220			
Observations	8527			

Statistics are z values, and p values in bold are lower than the conventional 0.05 threshold. Freq: frequency.

The error model above modelled all errors. However, we reasoned that internet speed may only have an effect on ‘no response’ errors, with the logic that if internet speed is slow then the picture may not display, and thus participants would provide no response. To test this, we removed all errors except ‘no response’ errors. A model with the same structure as above would not converge, thus we split upload and download speed into two separate models, and in each model we set the fixed effects to be both frequency variables and interactions between internet speed and each frequency variable (Table 7). For the upload speed model, we found a significant effect of word frequency and a marginally significant interaction between upload speed and phonotactic frequency. For the download speed model, we found a significant effect of word frequency, and a marginally significant effect of download speed.

**Table 7:** Generalised linear mixed model output on correct versus null responses, with separate models for upload and download speed predictors.

	Upload speed model				Download speed model			
*Predictors*	*Odds Ratios*	*CI*	*Statistic*	*p*	*Odds Ratios*	*CI*	*Statistic*	*p*
(Intercept)	15.66	9.69 – 25.28	11.25	**<0.001**	13.24	8.84 – 19.82	12.54	**<0.001**
Trial	1.08	0.99 – 1.17	1.73	0.083	1.05	0.98 – 1.13	1.35	0.178
Upload speed	1.19	0.77 – 1.85	0.79	0.427				
Word freq	1.28	1.04 – 1.58	2.29	**0.022**	1.27	1.05 – 1.55	2.40	**0.016**
Phonotactic freq	0.94	0.76 – 1.16	-0.60	0.551	0.92	0.76 – 1.12	-0.81	0.416
Upload speed*word freq	1.04	0.95 – 1.15	0.86	0.391				
Upload speed*phonotactic freq	1.09	0.99 – 1.20	1.80	*0*.*071*				
Download speed					1.39	0.97 – 2.00	1.80	*0*.*072*
Download speed*word freq					1.05	0.96 – 1.16	1.06	0.289
Download speed*phonotactic freq					1.04	0.95 – 1.13	0.78	0.437
**Random Effects**								
Residual variance	3.29				3.29			
Item intercept	2.04				1.82			
Participant intercept	1.80				1.52			
N	39 _participant_				49 _participant_			
	220 _item_				220 _item_			
Observations	6956				8566			

Statistics are z values. P values in bold are lower than the conventional 0.05 threshold, and italicized p values are marginal (between 0.05 and 0.1). Freq: frequency.

In general, we did not find any strong evidence of internet speed affecting whether participants were more likely to make an error overall, or whether internet speed affected whether participants provided a response or not.

## General discussion

We carried out an internet-based speech production study where participants overtly named pictures and audio responses were recorded via the internet. Our experiment was a replication of a lab-based experiment, giving us a baseline study to compare results to. We aimed to determine whether we could replicate the word frequency effect, and whether we would find evidence of the phonotactic frequency effect (which was not found in the lab study). We found strong evidence of the word frequency effect, and we found no evidence of the phonotactic frequency effect. This pattern of results follows the results found in the lab-based study. In addition, the size of the word frequency effect was the same between the internet and lab studies, even though participants’ speech production latencies were significantly longer in the internet experiment. We also found no compelling evidence that internet speed affected either participants’ response latencies or the proportion of errors they made. Finally, by exploring our data we determined that a sample size of 40 participants would suffice to detect an effect in an internet study. We discuss each of these points below, as well as challenges we faced in the implementation of the study and valuable lessons learned to pass on to speech production researchers.

### Word and phonotactic frequency effects

The word frequency effect is prevalent in the literature with average effect sizes of 40-50ms [13, 46, 47] and is robustly found in lab-based experiments. In contrast, the phonotactic frequency effect is less prevalent (several studies report fluctuating phonotactic frequency effects, finding significant effects under some experimental conditions, and an absence of effects under other conditions), with much smaller average effects sizes of around 10ms [31, 32, 48, 49] (with, to our best knowledge, as sole exception [12]). We found evidence here of a word frequency effect but no phonotactic frequency effect, likely due to the small effect size (9ms) and large standard deviation (around 30ms; see Table 5). Note the same results were found in the lab-based study, where no phonotactic effect was detected in the behavioural latencies (but an effect was detected in the ERP results). While it is possible that the experimental items in this study were not ideal enough to elicit the phonotactic frequency effect behaviourally, we have a strongly powered study with over 70 participants, yet the phonotactic frequency effect size we measure is in line with that found in the general literature. Thus, this suggests that evidence for the phonotactic frequency effect in the literature may be underpowered. Aside from this, the replication of our lab-based study demonstrates that speech production latencies can be reliably elicited online, despite concerns about accuracy and precision in timing.

### Internet-based versus lab-based latencies and error rates

While the size of the word frequency effect was similar between the two experiments, latencies were significantly longer by over 100ms in the internet study in all conditions. This could be due to technical recording issues, where the audio recordings launched earlier than picture presentation, adding extra time to the calculation of production latencies (see also [22] for similar reasoning). Error rates were also descriptively higher at 32% in the internet study compared to 22% in the lab study, and this could have been due to microphone issues resulting in empty recordings. While these technical issues are possible, we reinforce that the word frequency effect was still detected. Thus, for a within participant design, any technical timing or recording issues like these do not have a strong effect on the outcome. Another possibility is that we did not familiarise participants with the images they would see in the study, which can lengthen production latencies and result in a larger proportion of errors [26]. A final possibility, which we discuss more in the section *Ecological validity*, is that the lack of an experimenter being present removed pressure to quickly respond. There is some evidence that the presence of an experimenter can affect participant responses [50]. If the lack of an experimenter removed pressure to respond quickly, this may explain both why production latencies in this study were longer than those found in the lab, and why there were more errors (as people were either not responding or responding after the recording cut-off).

### Internet speed affecting responses

We hypothesized that participants’ internet speed, indexed by either upload or download speed, may have an effect on latencies and especially on error rates. However, we did not find compelling evidence for internet speed affecting latencies or errors. The only effects to reach significance were in the error analysis, where upload speed interacted with phonotactic frequency, where for low frequency items the higher the upload speed the more errors were given. This was likely driven by a spurious correlation between participants’ unique upload speeds and the few errors made. In addition, our error analysis comparing correct responses to null responses only showed marginal effects involving internet speed. Overall, we conclude that internet speed did not play a role in affecting the results found in this study, but we acknowledge that of our 71 participants only 39 provided internet speed data. We advise that researchers collect internet speed in their studies, as despite the lack of evidence here there are good logical reasons to expect that internet speed may play a role in response patterns. We collected internet speed by asking participants to visit a website (www.speedcheck.net) at the onset of the experiment, check their internet speed, and then report it. This is an easy and effective way to collect internet speed and then use it in further analysis.

### Ecological validity

One additional benefit of testing participants via the internet is that participants can carry out the experiment wherever they feel most comfortable, lending ecological validity to the manner of testing. For the majority of participants, data seemed to be collected while they were in their homes, based on the background noise in their recordings. For example, in some of the speech recordings, background noise included traffic noise, birds chirping, television noise, telephone notifications, and even other people’s conversations (the participants themselves were not involved in these conversations). Having this background noise initially led us to assume (while preprocessing the data) that we would not detect a frequency effect, as participants would be too distracted. However, even with this background noise, we still detected a word frequency effect comparable to that found in the lab. This is a strong advantage to internet-based testing, where we can demonstrate in a more ecologically-valid situation than the lab that psycholinguistic effects can still be detected. This has important implications for the word frequency effect as it is robust to distraction. However, this also gives us hope with testing other speech production paradigms, where internet-based studies may shed a light on how these classic effects translate to more ecologically-valid testing situations.

### Technical issues: Recording quality and data loss

In general, the quality of participants’ microphones was very good, and data quality was comparable to that of the lab. Data from only one participant needed to be excluded due to such low-quality recordings the words they produced could not be comprehended. However, more care would need to be taken if researchers wished to carry out a fine-grained phonetic analysis of collected speech. Our goal was to detect the onset of speech and transcribe what participants said. Whether the audio quality would be good enough for phonetic properties of speech to be discerned is an open question, and likely depends on the type of microphone participants used.

For seven of our participants, we ran into an unexpected error where the experiment terminated very close to the end of the experiment (approximately 10 trials from the end; range = 8 to 12 trials). These participants were using Firefox as their browser, and Firefox was recording audio responses in stereo with a large file size. This meant that the recordings in the experiment slightly exceeded the storage space per participant on the FindingFive server. This was fixed very quickly by the technical team at FindingFive, but was an error we had not anticipated encountering (nor had the team at FindingFive). This is an example of an unexpected error which can arise when testing on the internet. We encourage researchers to test their production experiments on a variety of browsers, or limit participants to take part only using one browser type, to avoid these kinds of errors.

### Bad-faith participants

Of the 100 participants tested, we had 22 ‘bad-faith’ participants. These were participants who let the experiment run on their computers to collect payment but did not actually do the experiment. They were easy to detect from their audio recordings, as they tended to be on the telephone, playing instruments, or singing along to music. We had not anticipated bad-faith participants as we recruited from our lab mailing list. However, there are multiple strategies to prevent bad-faith participants from taking part in or completing production studies. One method is to only give the experiment link and password to interested participants, and to make it mandatory for identifying information to be given at the at the beginning of the experiment. In our lab so far, this strategy has prevented participants from running though an experiment only for payment. Other possible prevention strategies include only paying participants once reviewing the data they provide, or building catch trials with incorrect responses resulting in termination. Bad-faith participants should not scare future researchers from running internet-based production studies, but they should be considered during participant recruitment.

## Conclusion

In conclusion, we carried out an internet-based picture naming experiment where we collected audio responses from participants. We were able to detect the word frequency effect in the collected data, and this effect was of the same magnitude as that found in a lab-based study using the same materials. However, production latencies were significantly slower when collected via the internet compared to in the lab. We additionally showed that internet speed did not affect production latencies nor errors, though we advise that researchers still collect internet speed information from participants. We also showed, from the data we collected, that a sample size of 40 participants would be ideal for a study of this kind to detect an effect. Finally, we discussed some of the lessons we learnt along the way in testing this novel methodology of speech production paradigms. We urge speech production researchers to deploy production experiments via the internet, both because the data collected are reliable and accurate, but also because participants are able to be tested in a more natural language setting. However, we think it prudent to first replicate multiple known effects to characterise the pattern of audio responses, before developing novel production methodologies.
